# A Novel AP2/ERF Transcription Factor CR1 Regulates the Accumulation of Vindoline and Serpentine in *Catharanthus roseus*

**DOI:** 10.3389/fpls.2017.02082

**Published:** 2017-12-06

**Authors:** Jiaqi Liu, Fangyuan Gao, Juansheng Ren, Xianjun Lu, Guangjun Ren, Rui Wang

**Affiliations:** Crop Research Institute, Sichuan Academy of Agricultural Sciences, Chengdu, China

**Keywords:** terpenoid indole alkaloids, RNA-seq, phylogenetic analysis, AP2/ERF, VIGS, qRT-PCR, metabolite analysis, *Catharanthus roseus*

## Abstract

As one type of the most important alkaloids in the world, terpenoid indole alkaloids (TIAs) show a wide range of pharmaceutical activities that are beneficial for clinical treatments. *Catharanthus roseus* produces approximately 130 identified TIAs and is considered to be a model plant to study TIA biosynthesis. In order to increase the production of high medical value metabolites whose yields are extremely low in *C. roseus*, genetic engineering combined with transcriptional regulation has been applied in recent years. By using bioinformatics which is based on RNA sequencing (RNA-seq) data from methyl jasmonate (MeJA)-treated *C. roseus* as well as phylogenetic analysis, the present work aims to screen candidate genes that may be involved in the regulation of TIA biosynthesis, resulting in a novel AP2/ERF transcription factor, CR1 (Catharanthus roseus 1). Subsequently, virus-induced gene silencing (VIGS) of *CR1* was carried out to identify the involvement of CR1 in the accumulations of several TIAs and quantitative real-time PCR (qRT-PCR) was then applied to detect the expression levels of 7 genes in the related biosynthetic pathway in silenced plants. The results show that all the 7 genes were upregulated in *CR1*-silenced plants. Furthermore, metabolite analyses indicate that silencing *CR1* could increase the accumulations of vindoline and serpentine in *C. roseus*. These results suggest a novel negative regulator which may be involved in the TIAs biosynthetic pathway.

## Introduction

Plants produce a vast number of secondary metabolites, when they adapt themselves to variable ecological environment during long-term evolution. Secondary metabolites are usually derived from primary metabolites and have their own unique metabolic pathways and different properties. They are generally classified into three major groups: phenols, terpenoids and alkaloids. As one type of the most important alkaloids in the world, terpenoid indole alkaloids (TIAs) show a wide range of pharmaceutical activities that are beneficial for clinical treatments (Van Der Heijden et al., 2004; Wasternack, 2014). Most TIAs are produced from the plants of Apocynaceae, Loganiaceae and Rubiaceae. One from Apocynaceae, *Catharanthus roseus*, produces approximately 130 identified TIAs and is considered to be a model plant to study TIA biosynthesis. Most of the TIAs produced by *C. roseus*, such as ajmaline, serpentine and catharanthine, show significant clinical medical values, moreover, vinblastine and vincristine, which are recognized to be the most valuable antineoplastic agents, have been widely used in the world. However, the yields of these high medical value metabolites are extremely low (Guéritte and Fahy, 2005).

During a long time, chemical synthesis, plant tissue, microbiological culture and other efforts have been done to increase the production of valuable secondary metabolites. But these methods still need to be optimized because of the excessive costs and low production. In recent years, genetic engineering has been applied in an attempt to increase TIAs yields, for example, transcriptionally regulating some specific genes in hairy root or cell suspension cultures to change the accumulations of specific TIAs (Papon et al., 2004; Runguphan et al., 2009; Peebles et al., 2011; Cui et al., 2015), while few great break-through have been made through these strategies. The major reasons could be: (1) few candidate genes involved in the accumulation of TIAs have been identified; (2) the limited effects on comprehensive TIAs synthetic pathway by single candidate gene. Hence, it is critical to find new candidate genes which could greatly facilitate TIAs production and the researches of TIA biosynthesis regulation.

Lots of studies suggest that genes in the TIAs synthetic pathway are under tight transcriptional regulations. It has been revealed that transcription factors are able to increase the production of TIAs via regulating the expression of multiple genes in the synthetic pathway. ORCA2 and ORCA3 are firstly identified AP2/ERF transcription factors in *C. roseus* which are involved in the TIAs synthetic pathway (Menke et al., 1999; Van der Fits and Memelink, 2000). Overexpressing either ORCA2 or ORCA3 in *C. roseus* suspension cells or hairy roots could change the expression of several key genes in the TIAs biosynthetic pathway (Van der Fits and Memelink, 2000; Pan et al., 2012; Li et al., 2013; Sun and Peebles, 2016). In addition, ORCA3 could be regulated by many inducers, such as jasmonic acid, artemisinic acid and fungal endophytes (Peebles et al., 2009; Pandey et al., 2016; Wang et al., 2016). Another two AP2/ERF members, ORCA4 and ORCA5, were cloned recently. They formed a cluster with ORCA3 and also can be induced by jasmonic acid, ORCA4 could act on the transcripts levels of genes in both tryptophan pathway and seco-iridoid pathway (Paul et al., 2016). In 2011, CrMYC2 in bHLH (basic helix-loop-helix) family, isolated by a yeast one-hybrid screening, could regulate the expression of ORCAs and also had a strong effect on the accumulation of catharanthine and tabersonine (Montiel et al., 2011; Zhang et al., 2011). WRKY transcription factor CrWRKY1 regulates several key genes in the TIAs synthetic pathway, especially TDC (tryptophan decarboxylase). Overexpressing CrWRKY1 in the hairy root of *C. roseus* caused significantly increased accumulation of serpentine (Suttipanta et al., 2011; Yang et al., 2013).

The genomic and transcriptomic information of *C. roseus* are not completely uncovered as many model plants like tobacco, Arabidopsis and rice. Hence, the studies aiming to excavate and functionally identify important transcription factors in *C. roseus* are not comprehensive. RNA sequencing is a widespread high-throughput technology in recent years (Mortazavi et al., 2008), which offers a holistic view of the transcriptome expression profiles of selected plant tissues or cells and provides an efficient way to identify transcripts involved in specific biological processes (Cloonan et al., 2008; Wilhelm et al., 2010). Therefore, excavating relative transcription factors combined with transcriptome sequencing data and understanding of the mechanism of transcriptional regulation during the overall TIAs biosynthetic pathway may promote our ability to break through the synthetic bottleneck (Aito et al., 2008; Saito and Matsuda, 2010; Guo et al., 2013; Verma et al., 2014; Yang et al., 2017).

In this study, three candidate AP2/ERF transcription factors were selected based on transcriptome sequencing data from *C. roseus* with methyl jasmonate (MeJA) treatment, and their responses to MeJA were verified by quantitative real-time PCR (qRT-PCR). After phylogenetic tree analysis of the three candidate genes with known AP2/ERF transcription factors involved in secondary metabolite biosynthetic pathway, CR1, which forms a cluster with OpERF2, was chosen for further study. Subsequently, Virus-induced gene silencing (VIGS) of *CR1* combined with metabolite analyses of silenced plants was performed to identify the involvement of CR1 in the accumulations of several TIAs. The results show that silencing *CR1* could increase the accumulations of vindoline and serpentine in *C. roseus*.

## Materials and Methods

### Plant Growth Conditions and RNA-Seq

Seeds of *C. roseus* cv. Rose Red were germinated in a greenhouse at 28°C, 16/8 h photoperiod on MS plates. After 4 weeks, seedlings with two to three pairs of true leaves were transferred to soil. Samples were collected from 1-month-old plants which were treated with MeJA for 0, 1 or 4 h. The MeJA experiment was with three replicates. Three plants were combined from each replicate time sample. MeJA treatment consisted of spraying 100 μM MeJA then placing plants under a clear plastic dome sealed with tape. Leaves of the plants were harvested and then frozen in liquid nitrogen.

Total RNA extracted from each material were subjected to cDNA library preparation and sequenced at Novogene Bioinformatics Institute (China) on Illumina HiSeq^TM^ 2000 sequencer (Illumina Inc., United States). Clean reads were aligned based on the reference genome (Medicinal Plant Genomics Resource, MPGR) (Kellner et al., 2015), transcript abundant estimation and expression level in fragment per kilobase exon per million mapped fragment (FPKM) were performed as described by Chen et al. (2017).

### Phylogenetic Analysis

Full-length amino acid sequences of candidate transcription factor as well as the AP2/ERFs which are potentially involved in secondary metabolite biosynthesis were aligned by ClustalW program. The neighbor-joining phylogeny was generated using MEGA 6 with bootstrap analysis of 1,000 replicates.

### Virus-Induced Gene Silencing

Seeds of *C. roseus* cv. Rose Red were germinated in a greenhouse at 28°C, 16/8 h photoperiod. Four weeks later, plants with two to three pairs of true leaves were used for VIGS. The genomic DNA of *C. roseus* cv. Rose Red was used as template to amplify *CR1* with specific primers (**Table 1**). The PCR-generated fragment was then introduced into the terminal vector pTRV2 by *Bam*H I and *Xho* I digestion. Catharanthus phytoene desaturase (PDS) involved in chlorophyll biosynthesis produces visible photo bleaching and serves as a useful marker for determining the best time to collect samples for testing. The purified plasmid DNA pTRV2-CR1, pTRV2 (negative control), pTRV2-PDS were respectively transformed into *Agrobacterium tumefaciens* strain GV3101 by electroporation. Three kinds of strains and GV3101 harboring pTRV1 were cultured overnight at 28°C in 300 ml Luria-Bertani medium containing 10 Mm MES, 20 μM acetosyringone and 50 μg/mL kanamycin. These bacterial pellets were collected and then resuspended in 5 ml infiltration buffer (10 mM MES, 200 μM acetosyringone and 1 mM MgCl_2_), and further incubated at 28°C for 3 h with shaking. The suspension of strain harboring pTRV1 was mixed with pTRV2-CR1, pTRV2 or pTRV2-PDS in equal volume just before the infection. The leaves of the seedlings were injected by a needle less syringe to fill the whole leaf with the bacteria, and each seedling was injected with 2–4 leaves. After injection, the plants were cultured in a constant temperature incubator. The PDS phenotype was observed 3 weeks after inoculation of leaves, and the other two sample plants were harvested at this stage. After recording the fresh weights of harvested materials, the samples were frozen in liquid nitrogen, one member of a leaf pair was used for RNA extraction, while the other was used for metabolite analysis.

**Table 1:** Polymerase chain reaction (PCR) primers used in this study.

Primer name	Primer sequence (5′ to 3′)^a^	Purpose
CR1-vigs-F	GGATCCGGATTATCAACAGGCTGATTC	Target fragment clone
CR1-vigs-R	CTCGAGGCAAGAGAGGCAAGGCATTT	Target fragment clone
CR1-F	GCTGCACTCAGGTTCAGAGG	Target gene in qRT-PCR
CR1-R	GCAAGAGAGGCAAGGCATTT	Target gene in qRT-PCR
CR2-F	CAAACCACTATACCCGCCGT	Target gene in qRT-PCR
CR2-R	GAAGAATCCGTTGCGTCAGC	Target gene in qRT-PCR
CR3-F	GCCGTCGAAAAGAAAAACCCA	Target gene in qRT-PCR
CR3-R	GCGGTGTCAAATGTTCCCAA	Target gene in qRT-PCR
STR-F	GCCTTCACCTTCGATTCAACTG	Target gene in qRT-PCR
STR-R	GTGGCTAGTTGTGTGGCATACC	Target gene in qRT-PCR
G10H-F	GCTCACAATCAATTCAAATTCTCCGT	Target gene in qRT-PCR
G10H-R	CAACCGCTTCTCCGCTCTGGCTATTT	Target gene in qRT-PCR
TDC-F	GGTCGAGGATGACGTGGCGGCCGG	Target gene in qRT-PCR
TDC-R	ACTCAGACTCAGTGAGTCAACTCGTT	Target gene in qRT-PCR
PRX1-F	GCGATTCATCAGTGCTGCTGGTGGGA	Target gene in qRT-PCR
PRX1-R	GTGGAAGGTTTGCTATTGTGTCTGCC	Target gene in qRT-PCR
SLS-F	CTTCACTCTTGAGAAACTAAAGTCAA	Target gene in qRT-PCR
SLS-R	AGTCAATTGTGAGATCCATGAGTT	Target gene in qRT-PCR
DAT-F	CTTCTTCTCATCACGTACCAACTC	Target gene in qRT-PCR
DAT-R	ATACCAAACTCAACGGCCTTAG	Target gene in qRT-PCR
SGD-F	CATTGGTGAACCGTGCTATG	Target gene in qRT-PCR
SGD-R	AGATTGTAGAGTCCAGATGGAACA	Target gene in qRT-PCR
RPS9-F	GCGTTTGGATGCTGAGTTGAAG	Endogenous reference gene
RPS9-R	GGCGCTCAAGGAAGTTCTCTAC	Endogenous reference gene

*^a^The underlined letters indicate the restriction enzyme sites.*

### Expression Analysis by Quantitative Real-Time PCR

Total RNAs of *CR1*-silenced plants and control were extracted and reverse-transcribed as described previously (Wang et al., 2015), and qRT-PCR was performed according to the same reference. The primers of *CR1, CR2* and *CR3* as well as 7 genes whose expression levels were detected in our work are shown in **Table 1**. Relative expression levels were determined using 2^-ΔΔC_T_^ method (Livak and Schmittgen, 2001). All qRT-PCR analyses were performed with three independent biological replicates.

### Metabolite Analyses

The selected samples were quickly pulverized in liquid nitrogen. Precisely weighed 0.3 g powder, mixed with 2 mL methanol, then subjected to extraction using the ultrasonic for 20 min, the supernatant was obtained after centrifugation. Repeatedly extracted with methanol for three times, then combined supernatant extract and compressed to dry, diluted to 2 mL with methanol, finally, filtrated with 0.45 μm microporous membrane before metabolite analyses by MS. The three standards catharanthine, vindoline and vinblastine were configured as a reserve solution of 10 ng/L with methanol, stored at -20°C, diluted to 10 ng/mL working fluid at the same time for testing purposes. ESI/MS/MS was used to detect the catharanthine, vindoline and vinblastine; using time-of-flight mass spectrometry (TOF-MS) to detect the serpentine.

## Results

### Three Candidate AP2/ERF Genes Were Selected Based on RNA-seq Data

**FIGURE 1:**
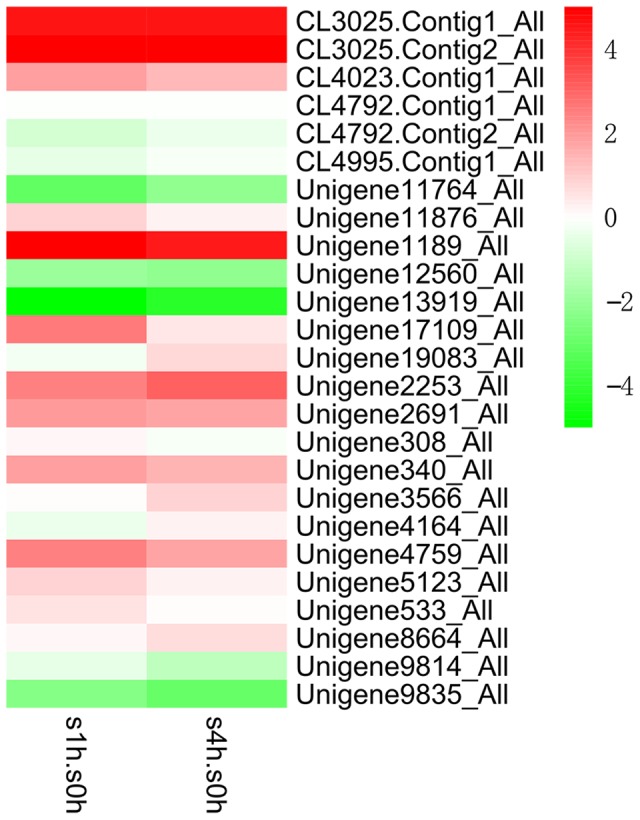
Expression patterns (log_2_ of FPKM) of AP2/ERF family in *C. roseus* at 1-h (s1h.s0h) and 4-h (s4h.s0h) after MeJA treatment. The expression levels are illustrated in green-red scale shown on the right. Green indicates lower expression and red indicates higher expression. Figure taken from Liu et al. (2017).

MeJA is identified as an important signaling molecule in TIA biosynthetic pathway in many plants (De Geyter et al., 2012). In order to excavate candidate transcription factors that may be involved in the regulation of TIA biosynthesis, we collected the leaves of 1-month-old *C. roseus* with MeJA treatment and extracted their RNA to generate the transcriptome sequencing data using Illumina platform. Subsequently, the expression profiles of different transcription factor families in *C. roseus* after MeJA treatment were analyzed. The expression profile of AP2/ERF family indicated that the expression levels of many AP2/ERF genes were changed obviously at 1-h and 4-h after MeJA treatment (their annotated gene names are shown in Supplementary Table S1). Three candidate AP2/ERF genes with different representative response to MeJA were selected for further studies (**Figure 1**). The expression level of Unigene9835_ALL (here designated as *CR1*) gradually decreased along the treatment. The expression level of Unigene2253_ALL (here designated as *CR2*) gradually increased after MeJA treatment. The transcript of Unigene1189_All (here designated as *CR3*) was up-regulated dramatically at 1-h and 4-h after MeJA treatment, while the expression level at 4-h was slightly lower than at 1-h. The expression pattern suggested that *CR3* is sharply induced by MeJA in a short time, and its expression level might gradually restore to the initial level. Above genes show different responses to MeJA treatment, and may be involved in the regulation of TIAs synthesis in *C. roseus*.

**FIGURE 2:**
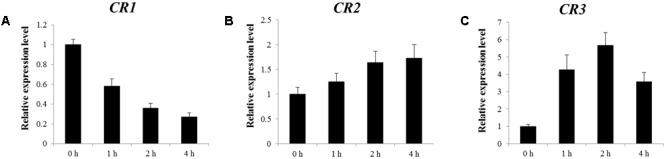
Relative expression levels of *CR1* **(A)**, *CR2* **(B)**, and *CR3* **(C)** responding to MeJA treatment. Figure taken from Liu et al. (2017).

In order to determine the accuracy of the RNA-seq data, the expression levels of three candidate genes at different time point after MeJA treatment were confirmed by qRT-PCR. **Figure 2** shows that after MeJA treatment, the expression level of *CR1* decreased gradually with time; *CR2* showed gradually increased transcript along MeJA treatment; the expression level of *CR3* was up-regulated rapidly and reached a peak at 2-h, then gradually declined. According to the results, expression patterns of three candidate genes under MeJA treatment were consistent with the RNA-seq data. Hence, the above candidate genes were subjected to further study.

### Phylogenetic Analysis

**FIGURE 3:**
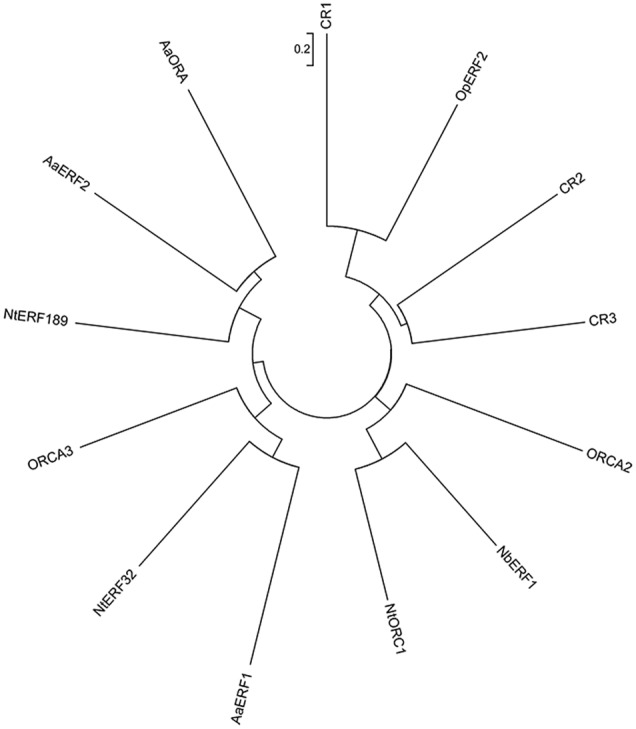
Phylogenetic analysis of three candidate AP2/ERF transcription factors and previously reported AP2/ERF transcription factors which are potentially involved in secondary metabolite biosynthetic pathway. ORCA2 (CAB93940.1), *Catharanthus roseus*; ORCA3 (ABW77571.1), *Catharanthus roseus*; AaERF1 (AEQ93554.1), *Artemisia annua*; AaERF2 (AEQ93555.1), *Artemisia annua*; AaORA (AGB07586.1), *Artemisia annua*; NbERF1 (ADH04266.1), *Nicotiana benthamiana*; NtERF32 (NP_001311965.1), *Nicotiana tabacum*; NtERF189 (NP_001312507.1), *Nicotiana tabacum*; NtORC1 (XP_016478305.1), *Nicotiana tabacum*; OpERF2 (LC171328.1), *Ophiorrhiza pumila*. Figure taken from Liu et al. (2017).

A phylogenetic tree was constructed based on the protein sequences of the three candidate transcription factors (for their sequences, see Supplementary Table S2), together with the previously reported AP2/ERF transcription factors which are potentially involved in secondary metabolite biosynthesis, to analyze their evolutionary relationships (**Figure 3**). CR1 was classified to the same group with CR2, CR3 and OpERF2. Besides, CR1 showed the closest relationship with OpERF2 which was identified recently to be involved in the regulation of specialized metabolism in *Ophiorrhiza pumila* (Udomsom et al., 2016), while CR2 and CR3 form the same cluster. The divergent clusters as well as the opposite response to MeJA between CR1 and CR2 or CR3 suggest the different molecular functions between CR1 and the other two. ORCA2 and ORCA3 involved in MIA biosynthesis in *C. roseus*; NbERF1, NtERF189 and NtORC1 regulating nicotine alkaloids biosynthesis; AaERF1, AaERF2 and AaORA regulating terpenoid biosynthesis in *Artemisia annua*, all of them showed more distant relationship with three candidate transcription factors than OpERF2 (Van der Fits and Memelink, 2001; Rushton et al., 2008; Shoji et al., 2010; Todd et al., 2010; De Boer et al., 2011; Yu et al., 2012; Lu et al., 2013; Thagun et al., 2016). Since CR1 showed a close phylogenetic relationship with other secondary metabolite-regulating AP2/ERFs, which suggested that CR1 may be a potential regulator in TIA biosynthesis, we selected CR1 for further functional characterization.

### CR1 Negatively Regulates 7 Key Genes in the TIAs Synthetic Pathway

Virus-induced gene silencing is a technique used for functional analysis of genes, which can be effectively applied in plants with long growth cycle and low conversion efficiency. The utility of this approach has been successful in exploring new genes that were involved in iridoid biosynthesis (Geu-Flores et al., 2012) and MIA biosynthesis (Liscombe and O’Connor, 2011) in *C. roseus*. In order to identify the function of CR1, we selected VIGS technology with the pTRV vector system (Dinesh-Kumar et al., 2003; Burch-Smith et al., 2004; Velásquez et al., 2009).

**FIGURE 4:**
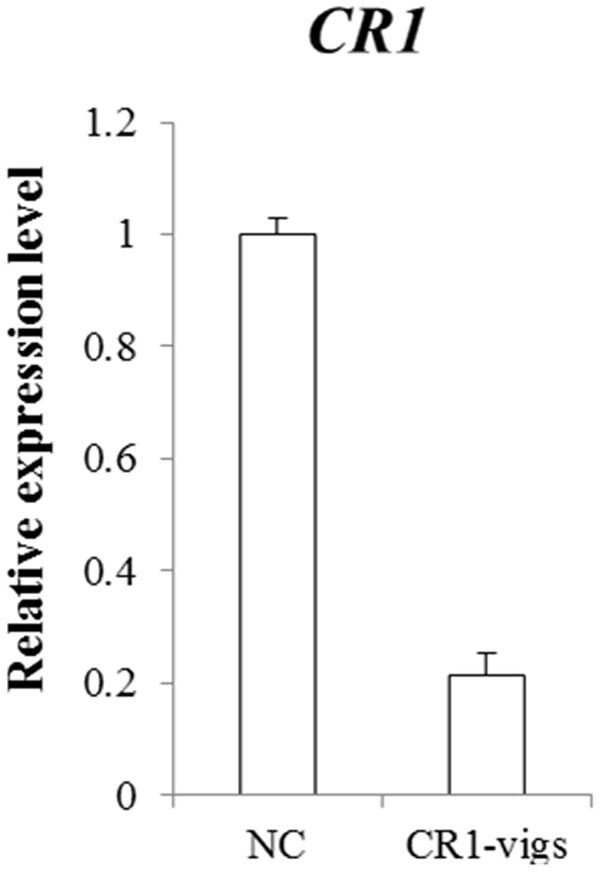
Effect of silencing *CR1* in *C. roseus*. The expression level of *CR1* in plants containing pTRV2-CR1 was detected by qRT-PCR to confirm the fold change of transcript levels from individual plants expressing the silenced gene compared to negative control (NC) containing pTRV2 empty vector. Error bars indicate standard error based on three independent biological replicates from three individual VIGS lines. Figure taken from Liu et al. (2017).

**FIGURE 5:**
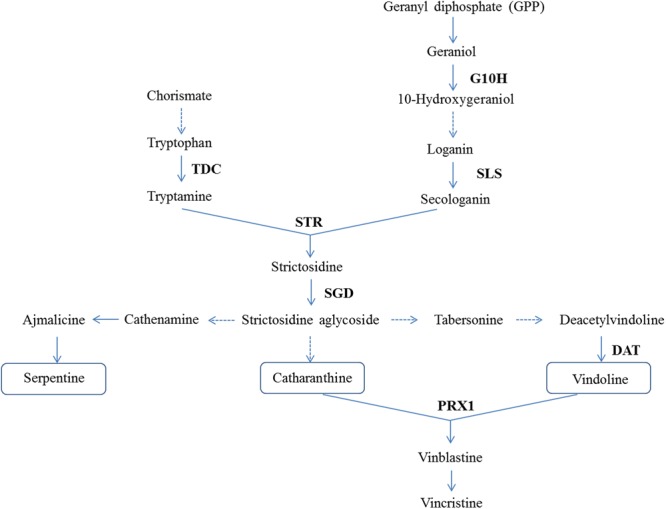
Seven genes (indicated in bold) which are representative in the TIAs synthetic pathway were subjected to expression analysis. G10H, geraniol 10-hydroxylase; SLS, secologanin synthase; TDC, tryptophan decarboxylase; STR, strictosidine synthase; SGD, strictosidine β-D-glucosidase; DAT, acetyl CoA: deacetylvindoline 4-O-acetyltransferase; PRX1, a-3′, 4′-anhydrovinblastine synthase. Figure taken from Liu et al. (2017).

**FIGURE 6:**
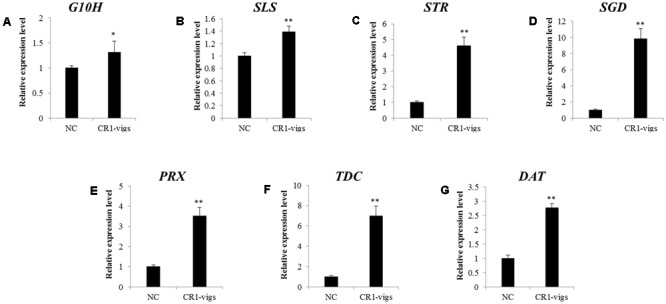
Relative expression levels of **(A–G)** *G10H, SLS, TDC, STR, SGD, DAT* and *PRX1* in TIA biosynthesis in *CR1*-silenced plants (CR1-vigs) compared to negative control (NC). ^∗^Indicates statistically significant differences (*P* < 0.05); ^∗∗^ indicates highly statistically significant differences (*P* < 0.01). Error bars indicate standard error based on three independent biological replicates from three individual VIGS lines. Figure taken from Liu et al. (2017).

Transcript analysis of *CR1* monitored by qRT-PCR revealed that the transcript level decreased by approximately 78% in *CR1*-silenced plants compared with the negative control (**Figure 4**). Subsequently, expression analysis of 7 genes (*G10H, SLS, TDC, STR, SGD, DAT* and *PRX*) which are representative in the TIAs synthetic pathway (**Figure 5**) in *CR1*-silenced plants was carried out. Their GenBank accession numbers are as follows: KF561461.1, L10081.1, X67662.1, X53602.1, EU072423.1, AF053307.1 and AM236087.1. The results indicated that silencing *CR1* in *C. roseus* could result in obvious changes in the expression levels of these genes. The expression levels of *G10H, SLS, TDC, STR, SGD, DAT* and *PRX* showed significant up-regulation (**Figure 6**). According to the previous results which suggest that the expression level of *CR1* is down-regulated in response to MeJA treatment, it can be inferred that CR1 may be a new negative regulatory transcription factor in the pathway of TIA biosynthesis.

### Accumulations of Vindoline and Serpentine Were Increased in *CR1*-Silenced Plants

**FIGURE 7:**
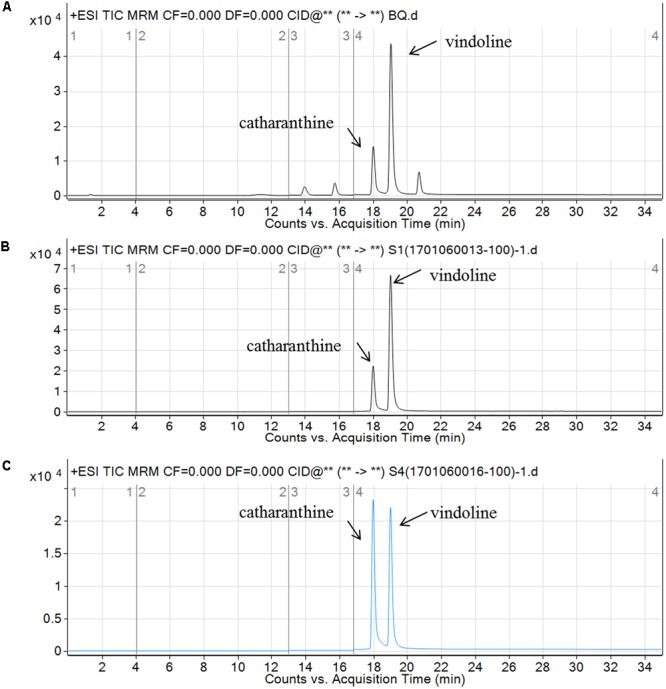
Vindoline and catharanthine component analysis in the leaves of *C. roseus* by ESI/MS/MS. **(A)** ESI Tandem Mass spectrogram for the vindoline and catharanthine standard; **(B)** vindoline and catharanthine ion current in the leaves of *CR1*-silenced plants shown by ESI/MS/MS. The two peaks are vindoline (m/z 457, RT 19.039) and catharanthine (m/z 337, RT 18.01); **(C)** vindoline and catharanthine ion current in the leaves of negative control shown by ESI/MS/MS. The two peaks are vindoline (m/z 457, RT 19.004) and catharanthine (m/z 337, RT 17.979). Figure taken from Liu et al. (2017).

**FIGURE 8:**
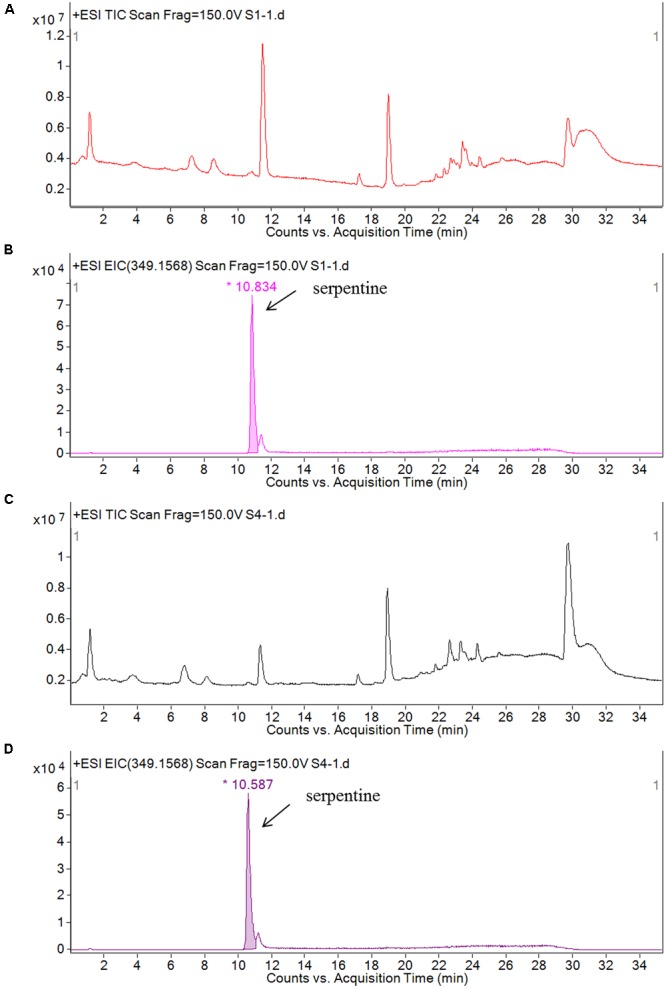
Serpentine component analysis in the leaves of *C. roseus* by TOF/MS/MS. **(A)** Total ion current in the leaves of *CR1*-silenced plants; **(B)** corresponding peak of serpentine (m/z 349 RT 10. 834) component in total ion current in the leaves of *CR1*-silenced plants; **(C)** total ion current in the leaves of negative control; **(D)** corresponding peak of serpentine (m/z 349 RT 10. 587) component in total ion current in the leaves of negative control. Figure taken from Liu et al. (2017).

ESI/MS/MS was used to detect the catharanthine and vindoline levels; time-of-flight mass spectrometry (TOF-MS) was applied to detect the serpentine. The accumulations of catharathine and vindoline in the leaves of *CR1*-silenced plants were 0.191 nmol/L and 0.153 nmol/L, compared to 0.198 nmol/L and 0.046 nmol/L in negative control plants (**Figure 7**). According to the results, silencing *CR1* could increase the accumulation of vindoline, but not catharanthine. Based on the peak area of serpentine detected by TOF-MS (**Figure 8**), we found that the accumulation of serpentine in *CR1*-silenced samples was higher than that in negative control. These results are consistent with the changes of the expression levels of related genes in *CR1*-silenced plants, which confirmed the inference that CR1 could regulate the synthesis of TIAs with negatively regulatory function in *C. roseus*.

## Discussion

It is well known that *C. roseus* which produces approximately 130 identified TIAs is considered to be a model plant to study TIA biosynthesis. Most of the TIAs produced by *C. roseus*, such as ajmaline, serpentine and catharanthine, show significant clinical medical value, moreover, vinblastine and vincristine, which are recognized to be the most valuable antineoplastic agents, have been widely used in the world. However, the yields of these high medical value metabolites are extremely low through traditional approach. Therefore, it is critical to find new ways which could greatly facilitate TIAs production, such as genetic engineering. Nowadays, bioinformatics has been widely used in many medicinal plants such as *Dioscorea nipponica* (Sun et al., 2017), *Panax ginseng C. A. Meyer* (Zhang et al., 2017), *Taxus chinensis* (Li et al., 2012; Meng et al., 2017), and *Ginkgo biloba* (Lin et al., 2011; He et al., 2015). High-throughput data such as transcriptome, proteome and metabolome could help us to gain an insight into the process of secondary metabolism in many non-model plants. The availability of several *C. roseus* transcriptomic databases, such as Medicinal Plant Genomics Resource (MPGR)^1^ (Kellner et al., 2015); PhytoMetaSyn^2^ (Facchini et al., 2012; Xiao et al., 2013) could greatly facilitate the studies on either biochemical pathways or transcriptional regulation in *C. roseus*. Our study utilized RNA sequencing combined with MPGR to compensate for the insufficiency of the genomic information as well as gene expression information in the discovery of candidate transcription factors involved in TIA biosynthesis regulation. By integrating bioinformatic analysis and a series of experimental methods, a novel negatively regulatory transcription factor, CR1, is identified.

The results of expression analysis of *CR1*-silenced plants suggested that CR1 could regulate several key genes in TIA biosynthesis, not only the genes upstream to the central precursor strictosidine which derive all the TIAs in *C. roseus* (G10H, SLS, TDC and STR), but the genes downstream to it (SGD, DAT and PRX1). ORCA2 and ORCA3 are the earliest found AP2/ERF transcription factors in *C. roseus* which play important roles in TIA biosynthesis. They mainly regulate the downstream genes in the seco-iridoid pathway (Menke et al., 1999; Van der Fits and Memelink, 2000; Pan et al., 2012; Li et al., 2013; Sun and Peebles, 2016), while ORCA4 is functionally overlapping but divergent with ORCA3, regulating the genes in both tryptophan pathway and seco-iridoid pathway (Paul et al., 2016). Combining these cases with our results, it can be inferred that CR1 may function together with other AP2 transcription factors as a negative feedback model to keep balance of TIA biosynthesis. Therefore, CR1 could be used in cooperation with other AP2/ERF such as ORCA3 to further increase the accumulations of target TIAs.

The suppression of *CR1* could promote the synthesis of vindoline and serpentine, but not catharathine, which may suggest that the genes in the catharathine-specific pathway can not be regulated by CR1. Vindoline and catharathine are the precursors of vinblastine and vincristine, the most valuable dimeric TIAs in *C. roseus* which have been used clinically to treat cancers since 1950s (Leveque and Jeh, 2007). If the accumulations of vindoline and catharathine are both increased, the biosynthesis of vinblastine and vincristine might be also promoted. Therefore, CR1 could be co-utilized with other transcription factors to increase the accumulations of vindoline and catharathine simultaneously. Overall, our study combined RNA sequencing and VIGS of the target gene as well as expression and metabolite analyses of silenced plants to discover an AP2/ERF transcription factor involved in TIA biosynthesis in *C. roseus*.

## Author Contributions

RW conceived and designed the experiments. JL and RW performed the experiments. RW and FG performed the data analysis. RW and JL wrote the paper. RW, FG, JR, XL, and GR revised the paper. RW secured the funds to support this research.

## Conflict of Interest Statement

The authors declare that the research was conducted in the absence of any commercial or financial relationships that could be construed as a potential conflict of interest.

## Footnotes

1 http://medicinalplantgenomics.msu.edu/

2 http://www.phytometasyn.ca/index.php/
