# Supplementary material for: A Novel AP2/ERF Transcription Factor CR1 Regulates the Accumulation of Vindoline and Serpentine in Catharanthus roseus
Source: Front Plant Sci. 2017 Dec 6;8:2082. doi: 10.3389/fpls.2017.02082 (PMC5724233; doi:10.3389/fpls.2017.02082)
Supplement: Supplementary file 1 [file Table_1.DOCX]

TABLE S1. Gene names of 25 Contigs/Unigenes of AP2/ERF family in RNA-seq data.

| **Contigs/Unigenes ID** | **Gene names** |
| --- | --- |
| Unigene9835_All | cra_locus_10963_iso_2_len_1010_ver_3 (CR1) |
| Unigene2253_All | cra_locus_9832_iso_1_len_1459_ver_3 (CR2) |
| Unigene1189_All | cra_locus_555_iso_10_len_1581_ver_3 (CR3) |
| CL3025.Contig1_All | cra_locus_20632_iso_2_len_765_ver_3 |
| CL3025.Contig2_All | cra_locus_15140_iso_2_len_1000_ver_3 |
| CL4023.Contig1_All | cra_locus_8562_iso_2_len_2136_ver_3 |
| CL4792.Contig1_All | cra_locus_11214_iso_4_len_1741_ver_3 |
| CL4792.Contig2_All | cra_locus_11214_iso_3_len_1934_ver_3 |
| CL4995.Contig1_All | cra_locus_22369_iso_1_len_1716_ver_3 |
| Unigene11764_All | cra_locus_13691_iso_2_len_999_ver_3 |
| Unigene11876_All | cra_locus_12447_iso_1_len_881_ver_3 |
| Unigene12560_All | cra_locus_16904_iso_1_len_826_ver_3 |
| Unigene13919_All | cra_locus_39038_iso_1_len_705_ver_3 |
| Unigene17109_All | cra_locus_5528_iso_2_len_956_ver_3 |
| Unigene19083_All | cra_locus_16657_iso_2_len_1928_ver_3 |
| Unigene2691_All | cra_locus_8217_iso_1_len_1874_ver_3 |
| Unigene308_All | cra_locus_21690_iso_3_len_447_ver_3 |
| Unigene340_All | cra_locus_15171_iso_2_len_896_ver_3 |
| Unigene3566_All | cra_locus_5474_iso_1_len_1799_ver_3 |
| Unigene4164_All | cra_locus_9969_iso_5_len_1155_ver_3 |
| Unigene4759_All | cra_locus_16027_iso_1_len_1313_ver_3 |
| Unigene5123_All | cra_locus_28505_iso_1_len_988_ver_3 |
| Unigene533_All | cra_locus_17242_iso_2_len_520_ver_3 |
| Unigene8664_All | cra_locus_10261_iso_3_len_1878_ver_3 |
| Unigene9814_All | cra_locus_12_iso_1_len_1626_ver_3 |
