# Supplementary material for: A Novel AP2/ERF Transcription Factor CR1 Regulates the Accumulation of Vindoline and Serpentine in Catharanthus roseus
Source: Front Plant Sci. 2017 Dec 6;8:2082. doi: 10.3389/fpls.2017.02082 (PMC5724233; doi:10.3389/fpls.2017.02082)
Supplement: Supplementary file 2 [file Table_2.DOCX]

TABLE S2. Amino acid and nucleotide sequences of CR1, CR2 and CR3 in this study.

| **Gene** | **Amino acid sequences** | **Nucleotide sequences** |
| --- | --- | --- |
| CR1 | MDWLMEPDHLQSSALTRENGVAEDQKLDEIIDIMNTSCLDKNTKDYQQADSFIDNTSGTVRRRYRGVRQRPWGKWAAEIRDPHKAARIWLGTFDTAEAAAIAYDEAALRFRGCKAKLNFPERVLLTSEVHAGSTSSSSKPNALPLLPDSFPDLFQYAMLLSSNDHELPPTSLLPKQIEQHQEEYACFTSRYLHTSSADASSLSETMKMKNKNQTEGCTLADVAVEDQSSIIVKAKYGDDMIKFELPLPSRKVKLEEEVKKRLKLSDGSFKIKYLDEDGDWILVACDVDLVNCIKVYKSLGQETIKMLVG | ATGGATTGGTTAATGGAGCCCGATCATCTCCAATCTTCTGCACTGACCAGAGAAAATGGTGTAGCTGAAGATCAAAAATTGGATGAAATAATTGATATTATGAACACAAGTTGTTTGGATAAGAATACAAAGGATTATCAACAGGCTGATTCTTTTATTGATAATACTTCAGGAACTGTCAGAAGACGTTACAGAGGAGTAAGACAAAGGCCTTGGGGAAAATGGGCTGCAGAAATTCGAGATCCCCATAAGGCTGCTCGAATTTGGTTAGGTACATTTGATACAGCTGAAGCTGCAGCCATTGCCTATGATGAAGCTGCACTCAGGTTCAGAGGCTGTAAAGCAAAGCTCAATTTTCCAGAACGCGTACTGTTAACAAGTGAAGTGCATGCTGGATCTACATCTAGCTCATCCAAGCCAAATGCCTTGCCTCTCTTGCCAGACAGTTTCCCTGATCTTTTCCAATATGCAATGCTACTTTCTAGCAATGATCACGAATTGCCACCTACATCTTTGCTACCGAAGCAGATTGAGCAACATCAAGAAGAATATGCTTGTTTTACCTCTCGATATCTGCACACTTCAAGTGCAGATGCCTCCAGTTTAAGTGAGACAATGAAGATGAAGAACAAGAATCAGACTGAAGGATGTACCCTGGCAGATGTAGCAGTAGAAGACCAGAGCAGCATCATTGTGAAGGCAAAATATGGAGATGATATGATAAAGTTTGAGCTTCCACTCCCATCTAGAAAGGTAAAATTGGAGGAGGAAGTAAAAAAGCGACTCAAGTTATCTGATGGGAGTTTCAAGATTAAATACTTAGATGAAGATGGTGATTGGATTCTAGTTGCCTGTGATGTAGATTTGGTGAACTGCATAAAAGTATATAAATCATTAGGCCAGGAAACAATTAAGATGTTGGTTGGTTAG |
| CR2 | MEKEENQTNKRVKYTEHRKQTTIPAVLTGRTRRLLPEMNGVVPKVVRISVTDADATDSSSDEDEVAAEAVFSRRQRVKRFINVVKIEPLCAGRDNGNGNGLSLNSGVLRSNNRSSTTADQNKKRKKSSGGANATAAKQTETDKVGGGNVKKFRGVRQRPWGKWAAEIRDPLRRVRLWLGTYDTAEEAAMVYDHAAIQLRGPDALTNFTTPPPKVSTPPPESTSSGYNSGDESHNDARSPKSVLRFVSSSNEDAEEESPHYEAVKESRECSRGGSDDVSVSENFSDYSLFPNDIFGFENPVSDFSDLFGGEGISAFEESSFDCGNTFIQSSTINDHMDFGFGSNPWPTTDDFFQDFGDIFGSDPLVAL | ATGGAGAAAGAAGAAAACCAGACCAATAAAAGAGTAAAATACACTGAGCACAGAAAACAAACCACTATACCCGCCGTACTAACGGGTCGGACAAGGAGGTTGTTACCGGAAATGAATGGCGTCGTTCCCAAAGTCGTTAGAATATCGGTGACGGACGCTGACGCAACGGATTCTTCAAGTGACGAAGACGAGGTGGCGGCGGAGGCCGTTTTTTCCAGACGCCAGAGAGTTAAGAGGTTTATTAACGTCGTTAAGATTGAACCATTGTGTGCTGGTAGAGATAACGGAAATGGTAACGGTTTAAGCTTAAACAGCGGCGTTTTGAGAAGTAATAATAGGTCGTCAACAACGGCGGATCAGAATAAGAAGAGGAAAAAAAGTAGTGGTGGCGCCAACGCTACCGCCGCAAAGCAAACGGAGACAGACAAGGTAGGAGGAGGAAATGTGAAGAAGTTTCGCGGGGTACGACAACGGCCATGGGGCAAATGGGCGGCAGAGATTCGTGATCCATTAAGACGTGTACGGCTATGGTTAGGCACTTATGATACTGCTGAAGAAGCTGCCATGGTTTACGACCACGCTGCCATTCAGCTGCGTGGACCGGACGCGCTTACTAATTTCACCACTCCTCCGCCGAAAGTGTCCACGCCGCCGCCGGAATCCACTTCCTCTGGTTACAACTCCGGCGATGAGTCTCATAACGACGCCCGTTCTCCTAAGTCAGTTCTCCGATTCGTTTCTTCTTCAAACGAAGACGCAGAGGAAGAGTCTCCTCATTACGAAGCCGTTAAGGAAAGCCGAGAATGCAGCAGGGGCGGAAGCGATGACGTGTCCGTATCGGAGAACTTCTCGGACTATTCGTTGTTCCCGAACGATATATTTGGGTTTGAAAATCCGGTATCCGACTTTAGTGACTTGTTTGGCGGAGAAGGAATTTCTGCTTTTGAGGAAAGTAGTTTTGATTGTGGAAATACGTTTATCCAATCGAGTACTATTAATGATCATATGGACTTCGGATTCGGATCAAACCCGTGGCCAACAACCGATGATTTCTTTCAAGATTTCGGTGATATTTTCGGGTCGGATCCTTTAGTAGCTCTTTAG |
| CR3 | MGSTTDEASTLEMITQLLLLDDFAFMEGYSVSNSSSSTSSSSTNDQSQTTTSSNSESISESDFNSSSITETSTFMAPTSFSFTCTNSNSAPESEYPQFFNPYPAINTGFNYFSQSQTNPQKISSSQSSKSPKFNERKPSLNIVPPPRKVLEFGKFNNNNFESTAVEKKNPDSEERRHYRGSDKGHGENSRRRFEIPTRKDPGFGWEHLTPPWKLLKLMTEPRLGLEGVKPY | ATGGGATCAACAACAGATGAAGCTTCGACTCTTGAGATGATTACACAGTTACTACTCCTTGATGATTTTGCATTCATGGAAGGTTACTCTGTTTCTAATTCTTCTTCTTCTACTTCTTCTTCTTCAACTAATGATCAAAGCCAAACTACTACTTCAAGTAATTCTGAATCCATATCTGAATCAGACTTTAATAGCTCTTCAATTACTGAAACTTCCACTTTTATGGCTCCCACATCTTTCAGCTTCACTTGTACAAATTCAAATTCAGCTCCTGAATCTGAATACCCCCAATTTTTTAATCCCTACCCTGCAATTAACACTGGATTTAATTACTTCTCTCAATCCCAAACAAACCCACAAAAAATTAGTTCATCCCAATCTTCAAAATCACCAAAATTCAATGAAAGAAAACCTTCCCTCAACATAGTACCTCCCCCGAGGAAGGTCTTGGAATTCGGGAAATTCAACAATAATAATTTTGAATCTACTGCCGTCGAAAAGAAAAACCCAGATTCGGAAGAGAGAAGGCACTATAGAGGCTCCGACAAAGGCCATGGGGAAAATTCGCGGCGGAGATTCGAGATCCCAACAAGAAAGGATCCAGGGTTTGGTTGGGAACATTTGACACCGCCGTGGAAGCTGCTAAAGCTTATGACAGAGCCGCGTTTAGGCTTAGAGGGAGTAAAGCCATATTGA |
